# How outsourcing has contributed to England’s social care crisis

**DOI:** 10.1136/bmj-2024-080380

**Published:** 2024-11-13

**Authors:** Benjamin Goodair, Adrienne McManus, Michelle Degli Esposti, Anders Bach-Mortensen

**Affiliations:** 1 Blavatnik School of Government, University of Oxford, Oxford, UK; 2 Department of Social Policy and Intervention, University of Oxford, Oxford, UK; 3 Institute for Firearm Injury Prevention, University of Michigan, USA; 4 Department of Social Sciences and Business, Roskilde University, Roskilde, Denmark

## Abstract

**Benjamin Goodair and colleagues** argue that growth of private provision in adult social care in England has resulted in worse care and should be rolled back

Adult social care in England is in crisis. Chronically underfunded services are struggling to accommodate unmet need, and inequalities are widening. The number of people applying and being rejected for care provision is rising year on year, and unmet need is twice as high in the most economically deprived areas compared with the least deprived.^1^ ^2^ Meanwhile, 9 in 10 adult social service directors in England did not believe there was adequate funding or workforce to meet care needs of older and disabled people in their area.^3^ These deficiencies have seen the social care sector brought “to its knees.”^4^

Care for older people and people with physical and mental disabilities is facing record demand but performing worse than any time in recent history. One contributor to this is the outsourcing of care provision to the private sector. Although competition from private sector provision was championed as a solution to achieve cheaper and better quality care, evidence from the past few decades in the UK and elsewhere challenges this view.^5^ ^6^ ^7^ In England, in particular, adult social care now faces a reality where reform is needed but the capacity for change is constrained by a model of care where most providers are run by for-profit companies.

## Commercialisation of care

Social care in England, sometimes referred to as community, residential, or personalised care, constitutes services that support people with activities of daily living and maintaining independence. In England, care services are largely divided between healthcare and social care, with local government responsible for organising and funding social care and the NHS a distinct service directly funded by central government. All health and social care services are regulated by the Care Quality Commission (CQC), an independent body responsible for inspecting, monitoring, and reporting on service quality.^8^

In England, healthcare is provided largely universally, whereas social care is means tested. A growing proportion of people do not qualify for state funded services and have to pay out of pocket because the threshold at which people have to pay for their own care has not been increased since 2010.^9^ The distinction between healthcare and social care was defined at the creation of the NHS in the 1940s, when services for those in “need of care and attention [but not] constant medical and nursing attention” were carved out of healthcare and designated to local authorities.^10^

The commercial interest in providing social care services has risen rapidly since the 1980s. Outsourcing—whereby the state pays private providers to deliver public service— was enabled by government legislation, in particular the decision to make social security grants available to residents in private care homes. Notably, this funding was not available for residents in public care homes and led to a boom in private (both third sector and for-profit) residential care.^10^ ^11^ This rise in private, but primarily for-profit, provision in the 1980s was accompanied by new regulation in the sector to avoid exploitation and low standards of quality.^12^

**Fig 1:**
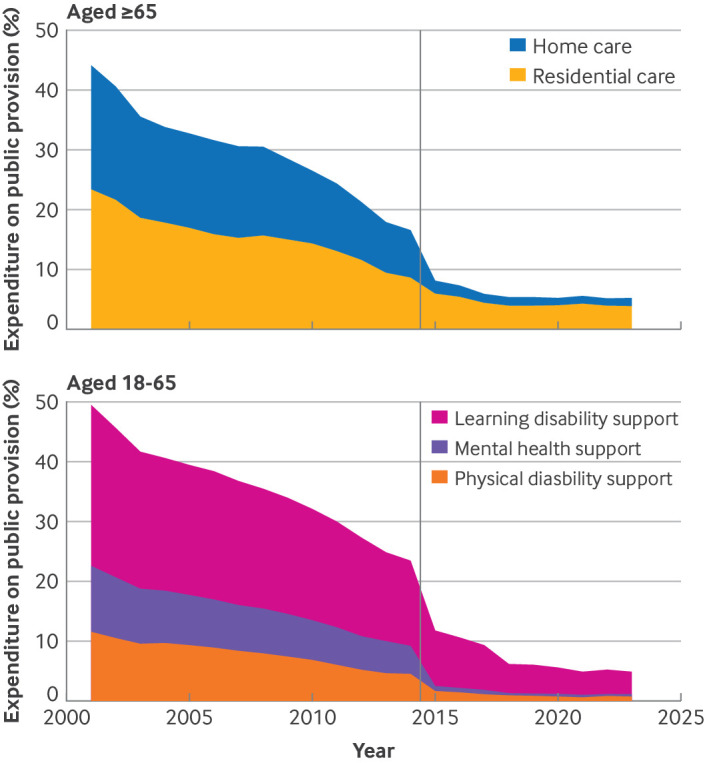
Local authority expenditure on public social care services in England, 2001-2023. Data collection processes changed in 2015 and spending before and after 2015 is not directly comparable.^14^ Spending on public services fell by 37.8% in 2001-14 and 29.2% in 2015-23. Taken together, we estimate a decline of 56% in 2000- 23

Outsourcing has continued to rise since the 1980s, and private provision of social care has steadily taken over. As a result, the public social care provision has all but disappeared and almost all services are provided by the private sector (box 1, fig 1). Extrapolation from the reported hours of care delivered by each sector suggests that 24 out of every 25 care residents are in private sector (for-profit and third sector) accommodation.^13^

Box 1 **Decline of public social care provision in England**

Published data that track a total of £194bn expenditure on services and a combined 279 million weeks of residential care provision from 2001 to 2023document how publicly provided social care has eroded and almost disappeared^13^:

- The average share of public services in local authority expenditure has declined from 40% to under 10%. In 2023 most local authorities spent nothing on public sector residential and home care services
- Data on residents in public provision corroborate the steep and radical decline in public service provision, decreasing from 27% in 2001 to 4% in 2023
- Although austerity measures saw large spending cuts to social care services after 2010, public expenditure had already substantially dropped by £300m between 2006 and 2010
- While there is large variation between geographical areas, most local authorities have seen declines in public provision over the past 20 years
- Most services are now run by for-profit providers with around 12% of care homes run by third sector organisations
- Social care services are now increasingly provided in the home, as housing support and community care. These too have been largely outsourced

## Why do services get outsourced?

Public services can be considered different degrees of “public” according to whether the government is in control of funding, provision, or regulation.^15^ Outsourcing refers to public services being delivered by privately owned organisations, including for-profit or non-profit third sector providers. Two theoretical arguments are commonly used to support outsourcing of public services. The first is aligned with the values of enhanced care provision whereby private providers, through adding a supplementary service, can offer different specialisms, capacities, and capital investment to the existing public service.^16^ ^17^ Core to this argument are assumptions about the behaviour of profit motivated providers. Profit motives in the private sector are assumed to make such providers more responsive to consumer needs, more willing to expand into new “markets,” and attempt innovation in how they deliver care.

The second argument rests on a more persistent ideological position about the value of competition, whereby the market is seen as the optimal provider of social goods. The argument is that the best service is achieved when service users become empowered consumers and service providers become competing vendors—and that such competition improves quality, reduces prices, and tailors services to residents’ needs.^18^ The best markets are considered to have a diverse and varied selection of providers, as this enables optimal competition and more service options for consumers. Following this argument, the main intention of allowing commercial provision is to build a mixed and diverse market of providers.^19^

In England, and elsewhere (box 2), both arguments have been used to motivate reform. The 1990s social care reforms under Margaret Thatcher’s government were widely justified by the idea that competition in a private market provides the most efficient services.^23^ This narrative has been pervasive. In the 2010s, legislation, white papers, and official policy documents aimed to create varied and mixed markets, and advocated for markets to provide “innovation, investment and continuous improvement” to service “efficient consumers.”^24^ Legislation in line with these aims paved the way to effectively eliminate publicly provided adult social care in England, based on the assumption that quality and value for money are protected, if not improved, in the process.

Box 2 **Public ownership of social care in Europe**

Between the mid-2000s and the mid-2010s, the share of care homes owned by private provision increased in almost every European country with available data:

- In Ireland, expenditure on for-profit home care grew from €3m in 2006 to €176m in 2019^20^
- 2015 legislative reforms in the Netherlands saw a large increase in for-profit nursing homes—doubling from 2015 to 2017 alone^21^
- The share of privately owned nursing homes increased in both Norway and Sweden between 2005 and 2014.^22^
- The proportion of private care homes rose to 35% in Slovakia, over 55% in Romania, and by 15 percentage points in Croatia^7^
- One notable exception, Cyprus, saw a growth in public care homes and a decline in private care homes between 2003 and 2014^7^

## Experience contradicts assumptions 

Research does not support the assumption that outsourcing of social care services improves quality.^5^ ^6^ Quality in social care requires ensuring the safety and wellbeing of care recipients. Observational studies have found that for-profit and private equity owned care homes deliver worse quality care than third sector or publicly owned homes.^5^ ^6^ ^25^ The outcomes are clear, but how do we know the comparisons are fair and that it is for-profit ownership causing this difference? It is not easy to make causal claims from observational studies, but the range and consistency of studies are compelling. First, quality differences are observed when private companies take over public services, suggesting that the same locations run by for-profit companies do worse.^26^ Second, these quality differences are observed in many countries and in different services, such as healthcare.^6^ ^27^ ^28^ For example, studies of covid-19 outbreaks and care home deaths in England, Canada, and the US found that, on average, more residents died after outbreaks which occurred in for-profit care homes than after those in public and third sector homes.^29^ This suggests that there is nothing unique to the context of adult social care services in England. And finally, the quality difference is observed in many different measures of quality, such as lower staffing rates or forced closures of care homes (an action of last resort when residents’ safety is at risk) suggesting that the for-profit gap is robust to different measures of quality.^30^ ^31^ Combined, there is prevailing evidence that the outsourcing of social care has not benefited residents, and with people’s safety at risk, there is sufficient cause for advocating changes to policy and regulation.^32^

Inequality has also been worsened as adult social care has turned to market based and more self-funded provision. A US study assessing the racial inequalities in covid-19 deaths, for example, found that nursing homes with higher rates of minority group residents “tended to be larger, for-profit, [and] chain-affiliated” and that these “for-profit nursing homes had 21% more covid-19 mortality.”^33^ Providers in England are increasingly focused on attracting affluent, self-funded, social care users, who pay higher fees than the rates set for state funded residents.^34^ This has led to services becoming less accessible in the most deprived areas.^3^ The end of public provision has meant that providers focus their commercial interests where the profit potential is highest. As a result, socioeconomically deprived people are now facing a double burden of service deprivation, while those in the richest parts of the country are more likely to have access to the care they need. 

Selective expansion of care provision has probably created issues of sufficiency: the number of care homes is falling, and the rate of unmet needs is increasing.^2^ Sufficiency and expansion of care capacity now relies on the private sector, but the financial incentives for providing social care are no longer linked to local levels of need.^35^

One reason for the failure of privatisation is that when quality is hard to measure, as it is in the care sector, market based provision is likely to incentivise cost cutting over quality improvements.^36^ ^37^ Commercial organisations are often most responsive to financial stimuli, especially as their survival in a market relies on profitability. Enforcing quality standards among private providers requires regulatory, contractual, or structural conditions that are difficult to implement. For-profit providers are therefore likely to maximise profits through cost reduction at the cost of quality, if regulatory and market structures allow them to.

The regulatory framework in England has proved ineffective at preventing the profit maximising behaviours that affect quality. This is partly because the primary role of the industry regulator (the CQC) is to measure the quality of services, and its enforcement powers apply to individual care homes performing below the regulation threshold rather than the underlying provider. It does not have any regulatory powers that can prevent the quality of care homes becoming worse on average, as long as homes are not performing below the enforcement threshold. More importantly, the CQC’s regulatory role has much less emphasis on statutory powers over provider chains and finances.^38^ ^39^ For example, even though the CQC has the powers to monitor the finances of social care providers, it merely operates an “early warning” system to local authorities once companies are at risk of failing.^40^ This light touch regulation means that profit seeking remains largely unchecked, allowing companies to cut costs and quality in pursuit of financial gain.

## Reducing the profit motive 

So how can we ensure that England’s ageing population and population with disabilities can access safe, equitable, and effective care? A partial solution is to control, restrict, or remove the profit motive in social care services, which would both improve the quality of provision and reduce inequalities across the system. This can be achieved in three ways. First, restricting the profit motive could be achieved by imposing additional regulation on social care providers. Examples of such measures include profit caps, limiting the payment of shareholder dividends, and restricting offshore and private equity investment and ownership. The downside of such measures is that they can be circumvented; evidence from the US shows nursing home companies using complex accounting techniques to hide profits between multiple companies owned by the same parent company or individual.^41^

A second option is attempting to align financial incentives with care quality through performance related payments. This approach faces multiple challenges. Quality is difficult to measure, and using the wrong metrics can lead to providers prioritising the targets at the expense of genuine quality, as observed in the NHS.^42^ Moreover, even with satisfactory quality measures, enforcement is difficult, and there is a risk of misreporting of self-reported data.^43^

A third option is changing the ownership of social care providers, bringing services back into public ownership or restricting all private ownership to third sector (non-profit) models. Because of the scale and embeddedness of for-profit provision in adult social care, a complete restructuring may not be feasible in the short term. Instead, incrementally commissioning local, small, ethical, and third sector provision while building up publicly owned capacity could be the first step in taking back control and gradually moving towards a care system less driven by the profit motive.^44^

Insufficient quality care can cause severe harm and distress for people who need it. Outcomes can range from people not receiving proper psychological support to preventable suffering, abuse, and death. Urgent steps to reduce the profit motive and reverse the outsourcing of services are essential to protect the growing population in need of care.

**Key messages**

The provision of adult social care services in England has almost been entirely outsourced to the private sector
The share of publicly provided adult social care has fallen by 56% since 2001
The increasing outsourcing of care provision has coincided with a care crisis and worse quality of care
Removing the profit motive would help improve quality and reduce inequities

## Notes

We thank the Wellcome Trust, Nuffield Foundation, and Carlsberg Foundation for financial support.

Contributors and sources: BG, ABM, AM, and MDE are academics, policy, and evaluation experts in public health, social policy, and epidemiology. BG and ABM specialise on the impacts of outsourcing health and social care services. AM has expertise on long term care policy in Europe. MDE has expertise in epidemiological evidence relating to social care provision. BG and ABM conceived of the initial idea. BG developed the first draft and curated the data. AM replicated the data and analysis. All authors contributed to subsequent drafts. BG is the guarantor.
